# Literary reading and eating disorders: survey evidence of therapeutic help and harm

**DOI:** 10.1186/s40337-018-0191-5

**Published:** 2018-04-16

**Authors:** Emily T. Troscianko

**Affiliations:** 1 The Oxford Research Centre in the Humanities (TORCH), University of Oxford, Oxford, UK; 2 Radcliffe Humanities, Radcliffe Observatory Quarter, Woodstock Road, Oxford, OX2 6GG UK

**Keywords:** Creative bibliotherapy, Eating disorders, Embodiment, Fiction-reading, Interpretation, Mental health, Self-help bibliotherapy

## Abstract

**Background:**

There is growing evidence for the efficacy of self-help bibliotherapy as a treatment for eating disorders, although little understanding of how specific linguistic characteristics may enhance or constrain its effects. Meanwhile, ‘creative bibliotherapy’ (the therapeutic use of fiction, poetry, or sometimes film, rather than self-help books) is widely practised, but even more poorly understood than the self-help variety: although a range of theoretical models exist, claims of the healing power of literature are far more commonly made than tested.

**Methods:**

An online survey including quantitative (forced-choice) and qualitative (free-response) items was designed and administered in collaboration with the charity Beat to investigate the connections between respondents’ reading habits and their mental health, with a focus on eating disorders, and attracted 885 respondents. Responses to two sequences of questions, exploring the differential effects of fiction about eating disorders versus respondents’ preferred genre of other fiction on the dimensions of mood, self-esteem, feelings about one’s body, and diet and exercise habits, were analysed using a 2 × 2 repeat measures factorial ANOVA design for each of the four dependent variables.

**Results:**

Surprisingly, fiction about eating disorders was perceived by respondents as broadly detrimental to mood, self-esteem, feelings about their bodies, and diet and exercise habits, while respondents’ preferred genre of other fiction was experienced as beneficial to mood and broadly neutral on the other three dimensions. The free-response data added detail to these core findings, as well as suggesting numerous other possible effects and mechanisms, drawing attention to the roles of positive and negative feedback structures and of highly selective interpretive filtering, and highlighting the dangers of ‘self-triggering’: using books to deliberately exacerbate an eating disorder.

**Conclusions:**

The findings directly challenge existing theoretical models of creative-bibliotherapeutic mechanisms, which tend to insist on the importance of a close match between the reader’s and the protagonist’s situations. They point the way forward for a new programme of clinical research and practice by suggesting other ways to conceive of how embodied cognitive acts of textually cued interpretation may intervene in the psychopathology of an eating disorder – for good and for ill.

## Plain English Summary

Self-help books are now commonly prescribed for many mental health conditions including eating disorders, and other books including fiction (e.g. novels, stories), poetry, and memoirs are often recommended by therapists or sought out by sufferers. But little is known about whether and how any kind of book can be therapeutically effective in managing or treating eating disorders. This article reports on an online survey involving nearly 900 respondents, which suggests that people with and without personal experience of an eating disorder perceive the books they read to be highly relevant to their mental health. In particular, respondents reported that fiction specifically about eating disorders (e.g. featuring a main character with an eating disorder) has negative effects on their mood, self-esteem, feelings about their bodies, and diet and exercise habits, while their preferred type of other fiction was seen as positive (especially for mood) or neutral. Some respondents also described deliberately seeking out particular books knowing that they were likely to make their disorder worse. Overall, these findings suggest an important role for reading in understanding, treating, and preventing eating disorders.

‘*Reading the Hobbit, of course, just makes one want food*.’ (anonymous survey respondent)

## Background

### Creative and self-help bibliotherapy

The perennial lack of resources for prevention and treatment of mental illnesses including eating disorders [1], combined with their biopsychosocial complexity, make ‘bibliotherapy’ – the reading of books, typically self-help books, for therapeutic purposes – an attractive option. Recommending that a patient read a particular book, or recommending that (s)he read more (or less) in general, has long been part of medical practice [2], but in recent years bibliotherapeutic approaches have begun to be formalised, and in the UK, for example, they are now commonly included in public health interventions [3], national healthcare trust provision [4], and clinical guidelines [5].

The efficacy of ‘self-help bibliotherapy’ (bibliotherapy using self-help manuals) has a growing evidence base in the treatment of eating disorders. A 2009 meta-analysis [6] found ‘promising’ effects for ‘first-step’ care in a range of studies, with slightly greater efficacy for guided self-help (supported by a nonprofessional) than for unguided or ‘pure’ self-help, but with high variability across the different eating disorders. These results were extended in 2013 with a further review noting an expanded evidence base for guided self-help in tackling behavioural symptoms of bulimia and binge-eating disorder [7]. A 2006 review [8] comparing pure and (professionally or nonprofessionally) guided self-help with other or no treatment found comparable effects to those achieved by other psychological therapies on bingeing and purging, other eating-disorder symptoms, interpersonal functioning, depression, and treatment dropout. This included moderate improvement for psychiatric symptoms and interpersonal functioning but no difference from waiting list on key behavioural criteria, and no overall difference between the two self-help types. However, most of the studies included in this review were small and probably underpowered.

All existing studies of bibliotherapy for eating disorders use self-help books based on the principles and methods of cognitive behavioural therapy (CBT), and all treat them as examples of CBT rather than as books: no linguistic details are ever mentioned, and even the titles tend to be buried in the references. Some consideration is given to the pros and cons of self-help, like lowering the barriers to seeking help, or working at one’s own pace, or conversely feeling a personal failure if recovery is not achieved [8–10], but these concern what it means to work alone as opposed to with a therapist, rather than to work with a specific text. Paradoxically, then, although the purpose of these trials is to assess the efficacy of self-help books, often in comparison with other therapeutic interventions, the books being used are to a large extent treated tacitly as neutral substitutes for a therapist: the distinct constraints, possibilities, and effects of their medium (written language) have not been subject to investigation in their own right. As Perkins and colleagues remark at the end of their review, more research is needed to establish what the ‘“active” ingredients in self-help treatment are’ [8, p.10].

To give one example of the kind of textual complexity that needs investigating: the self-help book used far more often than any other in the clinical research, Christopher Fairburn’s (1995) *Overcoming Binge Eating* [11], incorporates numerous (presumably fictional) vignettes from the perspective of a hypothetical eating-disorder sufferer (even including a ‘handwritten’ food diary and a supermarket receipt) alongside the factual information and behavioural and reflective tasks for the reader to complete. Yet the potential effects of this combination of textual features have been ignored in favour of a conception of the self-help manual in which written language is a transparent vehicle for delivery of CBT, not a richly selective and associative medium capable of generating a variety of specific (wanted and unwanted) responses in readers. If better understanding of the ‘active ingredients’ of textually mediated therapy is what we seek, research on the many forms of language use that make up the text in question will be crucial, and should not assume from the outset any hard dividing lines within generic or stylistic dimensions such as factual–fictional, narrative–discursive, literal–figurative, or concrete–abstract.

By definition, this point holds for any kind of text that may be enlisted therapeutically, whether ‘self-help’ or any other genre. Yet in part perhaps thanks to this neglect of the psychological capacities of language itself, genres which do not purport to deliver a direct alternative to face-to-face therapy have barely been researched in any context – and for eating disorders not at all. Rather more is known about the therapeutic practice of creative writing – variously known as poetry therapy, expressive writing, and therapeutic writing – for eating disorders, and this evidence is the subject of a recent review [12]. But here, I restrict my focus to the underexplored realm of reading. Although related processes of interpretation and sense-making are active in both, those involved in reading are both more prevalent as casual everyday activities than those of expressive writing; they are also structured by often extended engagement with a complex textual prompt, but often do not result in explicit interpretive ‘end products’ (beyond telling a friend or reflecting privately on what one thought of the book, for example). Hence reading and writing merit independent consideration. These features – the frequency of reading as an everyday habit and the qualities of specifically textually cued interpretation – will be explored further in what follows.

Despite its scarcity, what evidence there is for reading beyond self-help is promising. The most common term for the therapeutic use of books other than self-help is ‘creative bibliotherapy’. This tends to involve (guided or unguided) individual engagement with what we might call ‘literary’ works – prose fiction, poetry – but sometimes also includes film-viewing. Although the term arguably lacks transparency on first encounter, the findings of this study suggest that precisely its open-endedness – in particular not delimiting in advance the range of textual genres – is a significant advantage. A rare systematic review of causal evidence for creative bibliotherapy in the context of children’s behavioural development [13] found a small to moderate effect, and a more recent review for post-traumatic stress disorder [14] found promising trends in some low-quality and qualitative studies, though the authors located no high-quality controlled trials. Related work on reading groups rather than individually tailored reading has included observational qualitative studies finding marked benefits of engagement with novels, stories, plays, and poems for populations such as women prisoners [15] and people with depression [16]. No systematic evidence specifically for eating disorders exists at present, however.

Overall, as psychiatrist Jonathan Detrixhe puts it, ‘Unfortunately, thus far, the mere belief in fiction’s important place in the therapy room is held in higher esteem than the need to rigorously research the phenomenon’ [2, pp.60–61]. This holds both for the presence or absence of efficacy and for its possible mechanisms. Longstanding theories of creative-bibliotherapeutic change [17–19] tend to propose a process of *identification* (with the character in the text), *insight* (into the reality of the illness), and *problem-solving* [2], sometimes mediated by some kind of emotional catharsis. But these theories persist despite a paucity of supporting evidence beyond some case-study reports written by students [20] and very small-scale patient self-report data [18]. A partial exception is Zipora Shechtman’s [21] model, which involves exploring the problem, gaining insight, and committing to change, and which has some empirical support. But the model lacks theoretical precision as regards the materials used (stories, poems, and films are treated as equivalent) and the specific mechanisms of change involved (emotion and empathy apparently play a major, but underspecified, role). It is also noteworthy that all these theories are theories of guided self-help, with a professional helping the reader to tease out and apply what is valuable from the reading experience, but that the precise relations between the input from the text and from the therapist input remain fairly opaque.

Finally, Detrixhe’s comment also illustrates a general trend in the field to use ‘fiction’ as a synonym for ‘literature’, and thus as a catch-all for the kinds of texts used in creative bibliotherapy – implying that the factuality or otherwise of a text is the key dimension of relevance, and sidelining the potential importance of different dimensions, like narrative–discursive or prose–verse. Doubtless thanks in part to the influence of this tendency, the present study was designed in a way that makes the fiction–nonfiction distinction central, but as will become clear, the results suggest that it may in fact be something of a red herring. There is no perfectly neutral term to encompass all potentially relevant textual forms, but in what follows I will use ‘literary’ to denote all narrative and poetic textual genres (with no value-laden exclusion of contemporary, ‘popular’, or non-canonical, literatures) except where specific findings from the present study require references to ‘fiction’.

In addition to its privileging of fiction over other narrative or poetic forms, the dominant conception of the structure of creative-bibliotherapy-supported change involves many other assumptions about the type of text that should be used, including a range of value judgements about what constitutes suitably high-quality fiction [18], but most importantly the idea that the text should deal with characters and situations (specifically, problem/illness scenarios) as similar as possible to the reader’s own, and should provide detailed and realistic progressions towards a happy ending that resolves the problem [17]. No acknowledgement is made of the fact that reading a work of fiction (or other literature) written by someone else is different from, say, rereading one’s own diary, and that any benefits gleaned from such reading which differ from or exceed those of self-contemplation must therefore be due precisely to the gap between one’s own life and what is evoked in the text. When engagement with literary material is a source of self-directed insight, that insight comes, by definition, via engagement with people and worlds different (whether minimally or radically different) from oneself and what one knows. This also raises another unaddressed question: if we are aiming for as close as possible a ‘match’ between character and reader, which are the crucial dimensions of similarity? If suffering from the same illness as the character matters, does age or sex or sociocultural background or educational level matter too? If not, why not?

The classical model also does not allow for any possible value in a form of textually guided exposure therapy in which difficult experiences without happy endings could prompt a therapeutic confrontation with one’s own entrenched responses, followed by a gradual working-through to less dysfunctional patterns by continued engagement with flawed and complex textual characters. This possibility is raised by the beginnings of a CBT-inspired account which is otherwise broadly compatible with the tripartite structures characteristic of earlier theories [13]. This proposal suggests that the therapeutic reading process may involve recognition, reframing, identification, and emotional memories, but also makes space for the possibility that transportation into a textual world may permit critical engagement with entrenched posttraumatic responses that in the everyday world would elicit avoidance and distress [14]. That is, therapeutic fiction-reading need not exert its effects via neatly linear increases in insight and happiness.

In sum, the *identification–insight–problem-solving* schema dominates the field, but there are good reasons to question it on both empirical and conceptual grounds. For the eating-disorder context we might add another reason: the fact that in chronic eating disorders, levels of insight are often high but the ability to act on that insight very low. That is, seeking methods for heightening insight into one’s own condition by reading about people maximally similar to oneself may often be less valuable than encountering stimuli which through their very difference from one’s own reality generate a direct capacity for action, or indeed the belief that action could really lead to meaningful change.

We thus have several grounds for systematic research informed by attention to language as an elicitor of psychological change: 1) the first encouraging steps towards an empirical evidence base for therapeutic efficacy; 2) the sheer breadth of possibilities for fiction and other literary forms to expand one’s experiential horizons, from experiences of the fantastical and the sublime to access to the lifeworlds of the nonhuman and the extraterrestrial [22]; and 3) the fact that literature is without doubt already being used as a therapeutic tool, whether formally through institutions or more instinctively by individuals.

### Creative bibliotherapy and eating disorders

This paper builds on the theory and evidence presented thus far, taking eating disorders as a test case for the therapeutic relevance of textual genres other than self-help, and presenting the results of an exploratory scoping survey conducted in collaboration with the UK’s leading eating-disorder charity, Beat. But why eating disorders? The choice is not an arbitrary one: eating disorders are a particularly promising context for bibliotherapeutic research and practice for several reasons.

First, eating disorders are culturally inflected in salient ways, not least via the bodily ideals promoted in both image and text by online and offline media. On the anti-therapeutic side, pictorial depictions of the ‘thin ideal’ have been linked to women’s internalisation of this ideal, body dissatisfaction, and eating behaviours and beliefs [23], with conventional and social-media images potentially playing different roles [24]. Meanwhile, pro-anorexia websites exploit in varied ways the capacity of language to express and encourage psychopathology [25, 26], and beyond the realms of the body and illness, relatively homogeneous valuation of qualities such as self-control, self-denial, and restrictive notions of moral acceptability (especially for women) in post-industrial societies may also contribute to the development of value systems that feed disordered eating (whether or not to the point of a clinically diagnosable *eating disorder*).

Eating disorders have long been theorised in gendered terms as part of feminist discourse [27–29], but the development of feminist principles into treatment practices remains relatively rare [30]. That is, despite the general acceptance of a ‘biopsychosocial’ model of eating disorders, all three components are not treated equally [31] – and of course the ‘cultural’ is left only implicit within the ‘social’ category. But findings which suggest that prevalent cultural constructs can have detrimental impacts are a clear invitation to investigate the other side of the coin: whether different cultural inputs could have therapeutic effects. Literary texts may, of course, work hand in hand with other media. Conversely, however, linguistic and literary forms which do not replicate obviously constricting cultural norms have the potential to offer alternatives to inflexibly defined physical ideals and constrictive patterns of thought and behaviour – whether or not these relate directly to patriarchal forms of oppression.

In her book *Is Literature Healthy?* [32] Josie Billington suggests, for instance, that part of literature’s value is that it is the single area of our culture which seriously explores (and invites exploration) of the ‘inner life’. In *The Concept of Literary Application*, Anders Pettersson offers a nuanced model of the common readerly tendency to ‘apply’ literature to their own lives: through the qualities of concreteness (vividly evoking specific moments in time and space and experience), intentional design (giving the impression of being endowed with deeper meanings), openness (permitting multiple possible and appropriate interpretations), and a non-pragmatic context (not calling for immediate action), literature is, he suggests, ‘conducive to formation of nuanced perspectives on personally important matters in life’ [33, p.63] in ways that more abstract, shallow, closed, or pragmatic cultural forms are not. This model bears some relation to Russian Formalist (e.g. Shklovsky’s) and Czech Structuralist (e.g. Mukarovsky’s) theories of literariness as the capacity to induce changes in perception through foregrounding: through formal deviations from the norms of everyday language [34, 35]. In this model, without the formal disruptions characteristic of literary language use, cognitive change will not happen; with their help, anyone may potentially benefit from the shifting of habitual forms of thought and perception. Each of these theories has the beginnings of an evidence base, and all offer promising points of departure for investigating literature’s capacity to intervene in the culturally susceptible sphere of the eating disorder.

Secondly, eating disorders involve profound disruptions of embodiment: in particular, an over-emphasis on the numerical, visual, or otherwise externalised aspects of the body (size, weight, shape) at the expense of lived corporeality, often manifesting as a feeling of disconnection or disembodiment [36]. These disruptions always have cognitive components, but they are also always both effects and causes of changes in bodily states: shaped by and further exacerbating semi-starvation in anorexia, for example, or binge-purge/restrict cycles and the resulting bodyweight and endocrine fluctuations in bulimia. This means that simple behaviours which have a direct impact on the bodily state – eating more, exercising less, and so on – can make profound contributions to halting and reversing the progress of the illness. This in turn means that what is needed in the first instance is not the complex psychological work that might be required earlier on to tackle a condition like social anxiety disorder or even depression (though simple behavioural changes can obviously be powerful in those cases too); instead, what one needs is the motivation to change: to act differently; to eat differently. This requires at least some degree of belief that eating differently could make for a different kind of life, or a different way of understanding life – a belief that may be fostered by reading about people far removed from one’s own straitened circumstances. One possible facet of the therapeutic efficacy of reading literature may therefore, as mentioned in the previous section, be its potential to catalyse changes to attitude and thereby behaviour – whether or not through the generation of identification followed by the heightening of insight, as proposed in the well-known existing models.

A fundamental characteristic of the body-mind interactions that constitute eating disorders is the presence of self-perpetuating positive feedback loops which initiate the disorder and sustain it [37], and it is possible that cultural factors, including literary reading, may intervene in these loops for good or for ill: either heightening the instability or mitigating it. This capacity for culturally mediated effects may again be especially pronounced in eating disorders (and disordered eating more generally) because of the directness with which attitude can affect eating and eating then affect the whole mind-body system. This in turn is one of the most important arguments for incorporating any exploration of ‘cognitive’ manifestations of eating disorders (such as ‘body image’) into the broader context of interdisciplinary research on embodied (or ‘4E’: embodied, enactive, extended, and embedded) cognition [38]. The idea of combining both cognitive (e.g. neuroscientific) and embodied approaches with approaches focused on social and cultural factors is gradually gaining traction in eating-disorders research [39], and such rejections of polarised perspectives will be essential to more fully understanding any ‘mental’ illness.

Thirdly, literary texts, perhaps especially those that take narrative (as opposed to poetic or discursive) form, can be thought of as prompts to interpretive processes that have become disordered but are susceptible to therapeutic reconfiguration. Interpretation is a basic function of the human mind, and narratives can be both the vehicles and the products of interpretive activity. For example, the stories we tell ourselves about illness and health may take the form of self-generated, possibly non-verbalised narratives, which shape the experience of a given reality even as they concretise it [40]. Conversely, narrative structures created by others may provide the moulds by which our own interpretations are structured – or against which we rebel. These other-generated narratives may be as directly health-related as the taking of a medical history [41] or as broadly existential as a Modernist novel.

In eating disorders, the functions of interpretation can be seen as distorted at several points on a spectrum of over- to underactive interpretive engagement. At one end, as noted, sufferers may over-attribute significance to specific (often inappropriately externalised or objectified) body- and food-related stimuli [42, 43], and at the other, they may have avoidant attitudes towards the embodied experience of hunger that are predictive of clinical outcomes [44]. In the space in between, multiple and unstable shifts in body image can be elicited by over- or misinterpretations of perceptual, physical, emotional, and social cues [45]. As a complex prompt to interpretive processing at a remove from the raised stakes of immediately self-directed interpretation and from the narrow ideological agenda of much media output, literary texts have clear potential to intervene in the embodied pyschopathology of the eating disorder. The exploratory research presented here begins to establish how that potential may play out in practice, by asking people with and without a personal history of an eating disorder how they see the connections, if any, between their reading habits and their mental health.

## Methods

### Data collection

Our online survey was advertised on the research-participation section of the Beat website, via emails to Beat’s research volunteer database, on Beat’s monthly email newsletter, to Beat’s Young Ambassador cohort of volunteers, and through Beat’s blog and social-media (Facebook and Twitter) accounts. It was also publicised on the websites and social-media platforms of eating-disorder charities in Australia, Canada, the Republic of Ireland, and the USA, and via one personal blog (junealexander.com). To reach a broader population, the survey was also advertised on several email lists, webpages, and paper sign-up sheets around the University of Oxford, and a link was included on my personal email signature. The survey was restricted to respondents who self-reported an age of 18 or above, and was accompanied by country-specific information on where to turn for support with an eating disorder. All respondents read a downloadable information sheet, had the option to ask further questions, were aware that their participation was entirely voluntary, and gave consent for their data to be used anonymously in research publications unless they chose to withdraw consent at any later date.

The survey contained a total of 64 questions, although conditional branching created custom paths for individual respondents. We gathered basic demographic information, information about respondents’ experience of an eating disorder (if any), and details of respondents’ reading habits including favourite fiction titles and the three they had most recently read. (Note, again, that within the survey ‘fiction’ was the designator used, rather than any broader term like ‘literature’.) For respondents who reported personal experience of an eating disorder, a series of questions then followed concerning whether they had sought out fiction or nonfiction to help with their disorder and whether any books had been helpful or harmful, and if so how. Finally, to gain more detailed insight into the nature and intensity of responses to fictional texts on dimensions key to the eating-disorder psychopathology, a series of questions was posed concerning the self-reported impact of eating-disorder fiction, followed by the same series for the respondent’s preferred (and specified) genre of other fiction. (Preference was established via the question: ‘When you read fiction that is not fiction about eating disorders, which type of fiction do you usually read?’) The four dimensions chosen for this final series of questions were mood, self-esteem, feelings about one’s body, and diet and exercise habits. The survey concluded by asking respondents for any additional thoughts they might have on the subject of ‘your reading habits or how they relate to your mood, eating, exercise habits, or similar’.

For the purposes of this survey, ‘fiction’ was defined as ‘any non-factual material you read, such as novels, short stories, plays, or poems’. ‘Nonfiction’ was defined as ‘includ[ing] books like biographies and autobiographies, memoirs, textbooks, journalism, and other factual material’. ‘Fiction about eating disorders’ was defined as ‘fiction that includes one or more characters who have named eating disorders, and/or strongly exhibit the symptoms of an eating disorder, and/or exhibit any eating-disorder symptoms that are central to the story’. Eating-disorder fiction titles frequently mentioned by respondents included *Wintergirls* (Laurie Halse Anderson), *The Best Little Girl in the World* (Steven Levenkron), and *Girls Under Pressure* (Jacqueline Wilson). As prefigured above and explored in more depth below, our results suggest that the fiction–nonfiction distinction may not have been the one best suited to accurately capturing the patterns of respondents’ testimony. The tension between fiction and related categories (literature, narrative, prose) is analysed further in the concluding Discussion.

### Data analysis

Given the exploratory and observational nature of the study, responses to the majority of the forced-choice survey questions were analysed independently of any research hypothesis. After consideration of the data yielded in the rest of the survey (both forced-choice and free-response), two hypotheses were formulated for investigation of the four major dimensions of response (mood, self-esteem, feelings about one’s body, and diet and exercise habits), as follows:

1. Eating-disorder fiction (ED fiction) will elicit negative effects on the four dimensions specified; non-eating-disorder-themed fiction (non-ED fiction) will not.
2. Eating-disorder fiction will affect respondents with personal experience of an eating disorder more strongly than other respondents.

Consequently, respondents’ reports of the impact of reading two text types (ED fiction and non-ED fiction) on four dependent variables (Mood, Self-Esteem, Diet/Exercise, and Body) were analysed using a 2 × 2 repeat measures factorial ANOVA design for each of the dependent variables, with responses scaled from − 3 (major negative effect) to + 3 (major positive effect). Fiction type was the within-participants condition and presence/absence of a personal history of an eating disorder was the between-participants condition. The small number of respondents who had read ED fiction but had never had an eating disorder (*M* = 29.5) relative to the other categories (*M* = 246) meant that sample sizes were imbalanced. Nevertheless, the results were consistent with the hypotheses, and comparing random samples (*n* = 30) from larger groups against the smaller samples, as advocated in [46], confirmed the ANOVA model outcomes. Analyses were conducted in Python using the scipy, numpy, pandas, and statsmodels libraries, as well as SPSS 22.

Free-response data were subsequently revisited to glean further details about the quantitative findings, as well as to highlight key themes not manifest in the forced-choice responses and to offer alternative perspectives on the original research questions. Illustrative quotations (preserving typos and other idiosyncrasies) are presented in the following section.

## Results

The survey results indicate that reading accounts for a significant proportion of many respondents’ weekly habits, and is perceived to have significant effects – negative, positive, and neutral – on factors related to disordered eating. These effects were reported both by those who reported personal experience of an eating disorder and by (the much smaller group of) those who did not. In particular, a striking difference emerged between the effects of reading fiction about eating disorders and respondents’ preferred type of other fiction: the former was reported as broadly negative in effect and the latter as broadly neutral or positive. Free-response data shed further light on this and other phenomena of textual response.

**Fig. 1:**
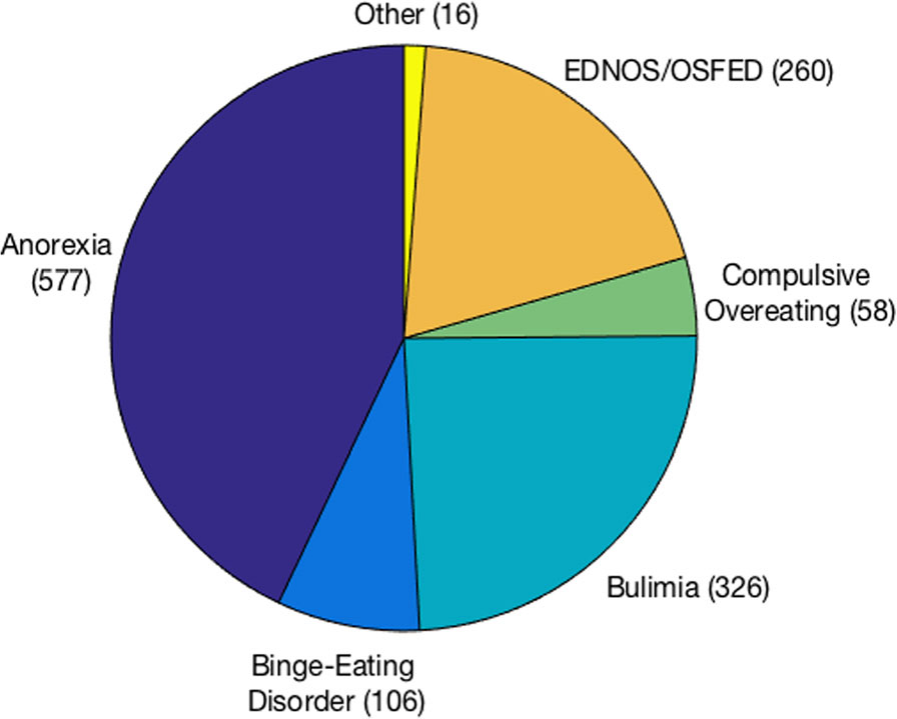
Reported eating-disorder types

The survey was live for 12 weeks, and in that time it attracted 885 respondents (completing all questions was not compulsory, and 48 additional respondents were excluded whose responses ended at or before Q4), the majority through the UK version of the survey. The respondents were predominantly female (847 female, 32 male, 6 not specified) and in their 30s or under (*M =* 28, range 18–75, *SD* 10.26). 773 reported personal experience of an eating disorder (of whom 749 were female, 20 male, 4 not specified; *M* = 27, range 18–71, *SD* = 8.97). Respondents were free to report experience of as many eating-disorder types as necessary, and a total of 1343 were reported (see Fig. 1). (In the ‘other’ category, respondents specified conditions including laxative abuse, orthorexia, diabulimia, and compulsive exercise.)

**Fig. 2:**
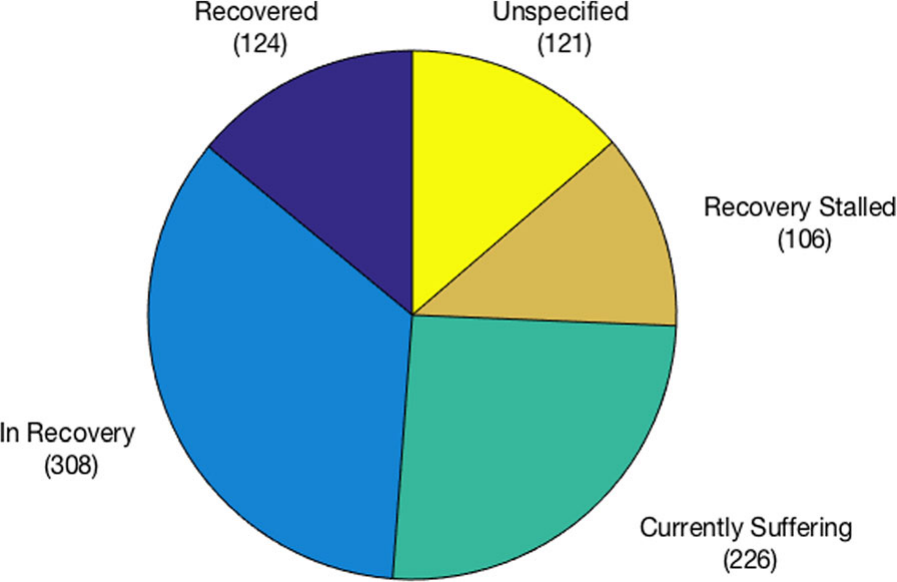
Reported status of illness or recovery

Of the 112 respondents who reported no personal experience of an eating disorder, 51 reported having a family member with a past or present eating disorder (the majority a child or sibling). The most commonly reported age of eating-disorder onset was 14, and of the 716 respondents who reported the age of onset of their disorder, 95% reported onset at or under 22 years of age (*M* = approx. 15, range: under 11–56). Respondents were asked to select from a list their recovery status at the time of participation (see Fig. 2). (The ‘recovery stalled’ category denotes those who had been in recovery but whose recovery had ‘stalled before I became fully healthy’.)

Respondents reported substantial time spent reading per week, and there was no significant difference between reported time spent reading amongst respondents overall versus the subgroup of respondents with personal experience of an eating disorder: in both, 28% reported less than 1 h per week, 48% between 1 and 5 h, 15% 5–10 h, and 9% more than 10 h. The traditional book was still reported as the most commonly used medium, but ereaders and tablets were also popular. Only 8% of respondents reported membership of a reading group or book club. Classic or literary fiction was the most popular fictional genre overall, for respondents with and without experience of an eating disorder, but crime and detective fiction, fantasy, and young-adult fiction were also popular with both groups. The other categories we included were: comedy/humour, comics / graphic novels, drama (i.e. theatrical plays), erotic fiction, experimental fiction, historical fiction, poetry, romantic fiction, science fiction, and suspense/thriller. Amongst respondents with a history of an eating disorder, the least popular genres were comics / graphic novels, drama, erotic fiction, experimental fiction, and poetry, each of which was mentioned by between only 3 and 6 respondents. (These genres were also infrequently read by those without experience of an eating disorder.) All other genres were selected at least 16 times (3%) by those with an eating-disorder history, with fantasy at 11% (52 mentions) and classic/literary fiction 23% (106). In the ‘other’ category, respondents mentioned genres like ‘modernist/philosophical’, ‘psychological novels’, ‘supernatural’, ‘family drama’, and ‘fanfiction’, as well as others that would not normally be considered fiction, like ‘biographies’ or ‘social media / websites’. Some respondents also used the ‘other’ category to list several types as equally often read. Respondents’ recently read and favourite books and authors covered a vast range of genres and periods, but the two names that recurred much more often than others in the ‘favourites’ list were J.K. Rowling (32 mentions) and Jodi Picoult (27); both had correspondingly many mentions in the ‘recently read’ category too, but classics by Tolkien and Austen, as well as modern ‘literary’ fiction by Ian McEwan and fantasy by Terry Pratchett, also recurred frequently in this category.

One of the broad trends observed in reported responses to fiction and nonfiction was the fact that fiction is often turned to as a source of support for eating-disorder sufferers (see Table 1).

**Table 1:** Reported uses of fiction and nonfiction

Reported uses of fiction (F) and nonfiction (NF)	Percentage of respondents with personal history of an eating disorder (*n* = 773)
Looked for F or NF to help someone else with an eating disorder	31% (of whom 29% found F or NF to recommend)
Looked for F or NF to help with their own eating disorder	69% (34% had done so often)
Had F or NF recommended by someone else to help with their eating disorder	50% (40% tried reading the recommended text)
Found F or NF helpful with respect to their eating disorder	36%

Helpfulness rankings of different text types (on a 5-point scale) by those with personal experience indicate that eating-disorder self-help books are judged the most helpful type of book, followed by biography/autobiography/memoir about eating disorders, followed by other nonfiction, followed by fiction about eating disorders and then other fiction (see Table 2 for aggregated ratings). For other fiction, 15% of those who ranked it as having been helpful ranked it higher than all other text types.

**Table 2:** Helpfulness and harmfulness of main text types

Text type	Average helpfulness score	Average harmfulness score
ED self-help	87% (*n* = 331)	51% (*n* = 207)
ED biography/autobiography/memoir	77% (*n* = 296)	73% (*n* = 298)
Other nonfiction	53% (*n* = 201)	35% (*n* = 143)
ED fiction	50% (*n* = 190)	64% (*n* = 258)
Other fiction	44% (*n* = 169)	32% (*n* = 128)

Rating the perceived harmfulness of textual engagement in tackling their eating disorder, respondents ranked eating-disorder fiction and eating-disorder biography/autobiography/memoir significantly higher than the other text types (Table 2). Meanwhile, other fiction was ranked less harmful than all other text types by 20% of those who ranked it.

**Fig. 3:**
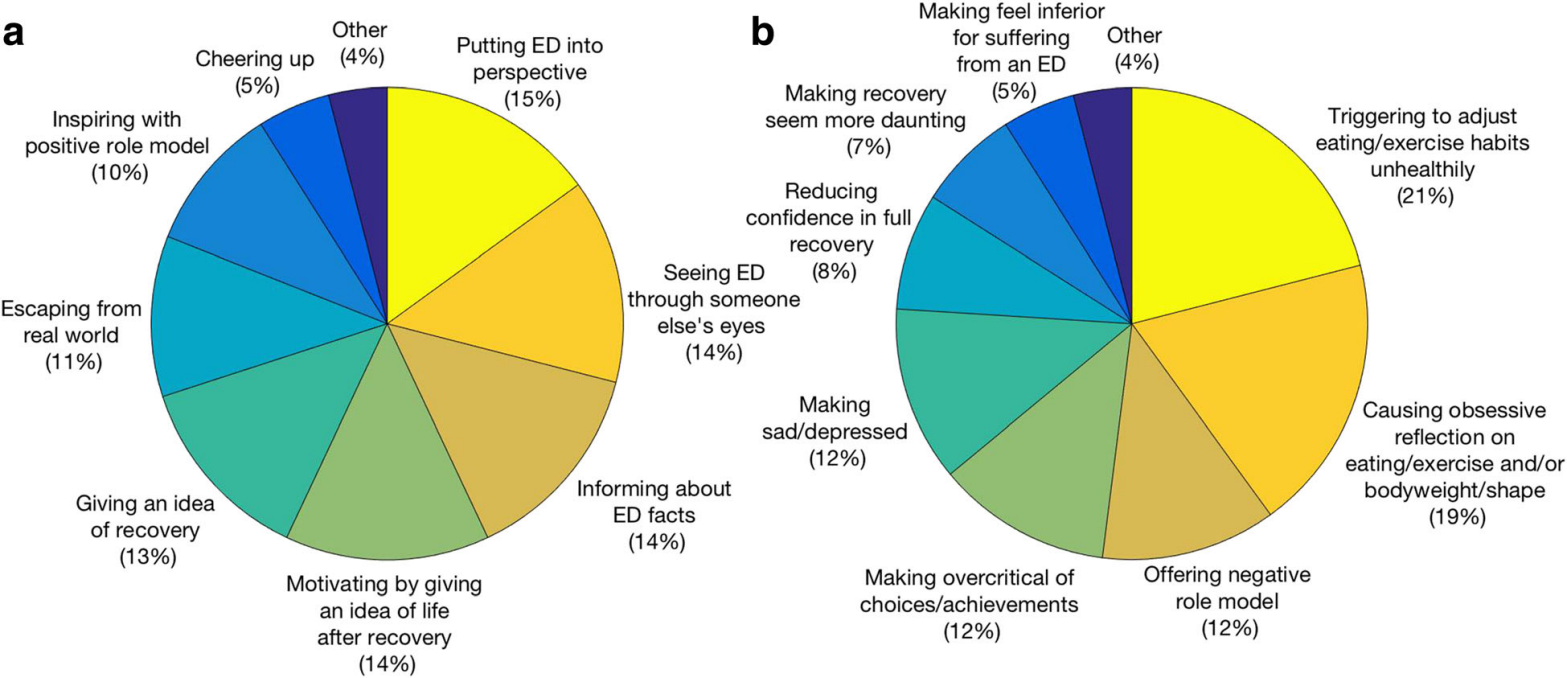
**a** and **b** Reported mechanisms of textual **a**) helpfulness and **b**) harmfulness (for fiction and nonfiction). In the final survey, two near-identical items, ‘causing obsessive reflection on eating/exercise’ and ‘causing obsessive reflection on eating/exercise and/or bodyweight/shape’, were mistakenly retained in the question about mechanisms of harmfulness. Both were selected by 16% of respondents, and the former has been omitted here for clarity

Subsequent questions explored by what mechanisms help and harm may be achieved. Referring to both fiction and nonfiction, there was a relatively even spread of reported effects across the full range of options we provided (Fig. 3a and b). Helpfulness indicators suggest a particular role for gaining more or a different perspective on illness and recovery, while harmfulness may be mediated in particular by changes to eating disorder-related behaviours and an increase in obsessive thoughts about those behaviours or bodyweight and shape. Suggestions frequently raised in the ‘other’ categories included the harmful role of competitive comparisons between self and textual other and the helpfulness of simply knowing one is not alone.

**Fig. 4:**
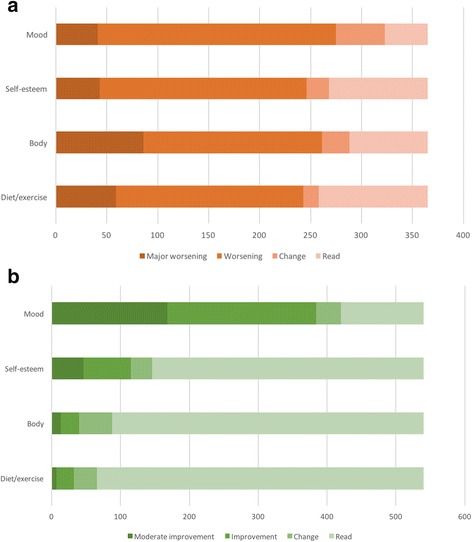
**a** and **b** Dimensions of response to **a**) fiction about eating disorders and **b**) respondents’ preferred genre of other fiction. These charts indicate how many respondents reported having read the two fiction types (ED fiction 365, other fiction 540), and within that cohort, how many reported experiencing any change on the dimension in question, then how many reported improvement or worsening, and then how many reported minor, moderate, or major improvement or worsening. Each category is subsumed within the previous one: e.g., for ED fiction and diet and exercise habits, 258 of the 365 who had read ED fiction reported experiencing some change, 243 of those 258 reported a worsening, and 59 of the 243 reported major worsening. For readability’s sake, the most striking effects are illustrated here (i.e. major negative responses to ED fiction and moderate positive responses to other fiction). Both analyses included responses from all 885 respondents. Analysis of the responses to other fiction was also performed solely for the subset of respondents who reported having read ED fiction, in case this group also responded differently to other fiction types, but the pattern of responses was not significantly different from those manifested by the wider group, shown here

When it came to the effects of reading eating-disorder fiction or other fiction on mood, self-esteem, feelings about one’s body, and diet and exercise habits, a striking contrast emerged between the two text types. Eating-disorder fiction was associated with overwhelmingly negative effects on all four dimensions (see Fig. 4a). For respondents’ preferred type of other fiction, the picture was entirely different, with the dimension of mood manifesting the clearest, and most positive, pattern (see Fig. 4b). These findings make clear that all four dimensions are likely to be highly relevant in further exploration of mechanisms of text-cued therapeutic and anti-therapeutic change, with mood especially salient on the positive side.

Statistical analysis of the impact of reading eating-disorder fiction versus respondents’ preferred genre of other fiction on these four dimensions showed significant effects of fiction type. Broken down by dimension, the results were as follows.

**Fig. 5:**
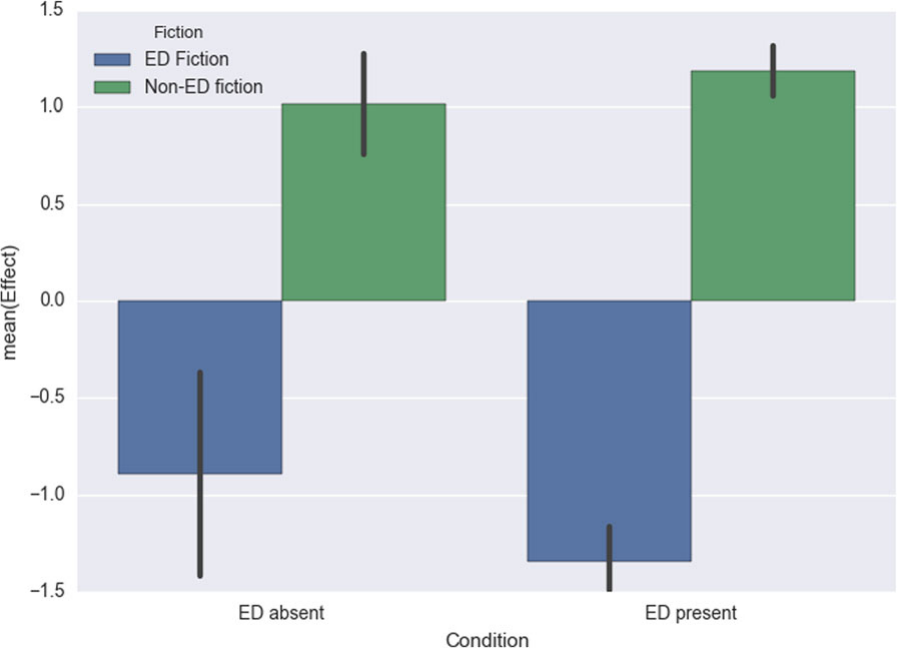
Impact on mood by fiction type and presence/absence of personal eating-disorder experience

Testing for Mood showed a significant main effect for fiction type (*F*(1, 257) = 118.967; *p* < .001, *d* = 1.588) in the predicted direction, with respondents evaluating non-ED fiction effects as more positive (*M* = 1.148; *SD* = 1.022) than those of ED fiction (*M* = − 1.309; *SD* = 1.284). As per Cohen [47], the effect size – calculated as Cohen’s *d* – can be classed as large. There was a non-significant effect for ED condition (*F*(1, 257) = 0.855; *p* = .356). The interaction between fiction type and ED condition (present or absent) was not significant, with *F*(1,257) = 2.608 and *p* = .108 (Fig. 5).

**Fig. 6:**
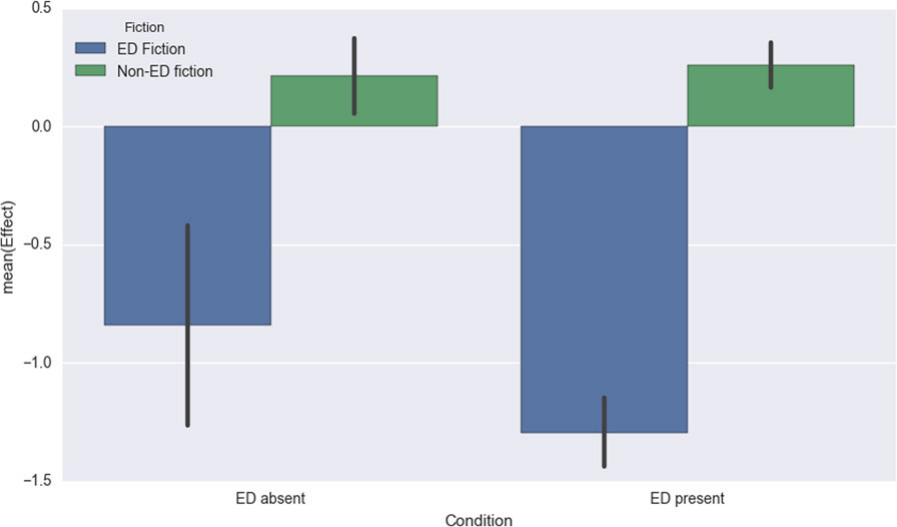
Impact on self-esteem by fiction type and presence/absence of personal eating-disorder experience

Results for Self-Esteem revealed a significant main effect of fiction type with large effect size (*F*(1, 266) = 62.520; *p* < .001; *d* = 1.165) and no main effect for ED condition (*F*(1, 266) = 0.317; *p* = .574). The significant main effect for fiction type was in the expected direction, to the extent that ED fiction had a much greater negative impact on self-esteem (*M* = − 1.263; *SD* = 1.163) than non-ED fiction (*M* = 0.25; *SD* = 0.745). The interaction between fiction type and ED condition was not significant: *F*(1, 266) = 3.740; *p* = .054 (Fig. 6).

**Fig. 7:**
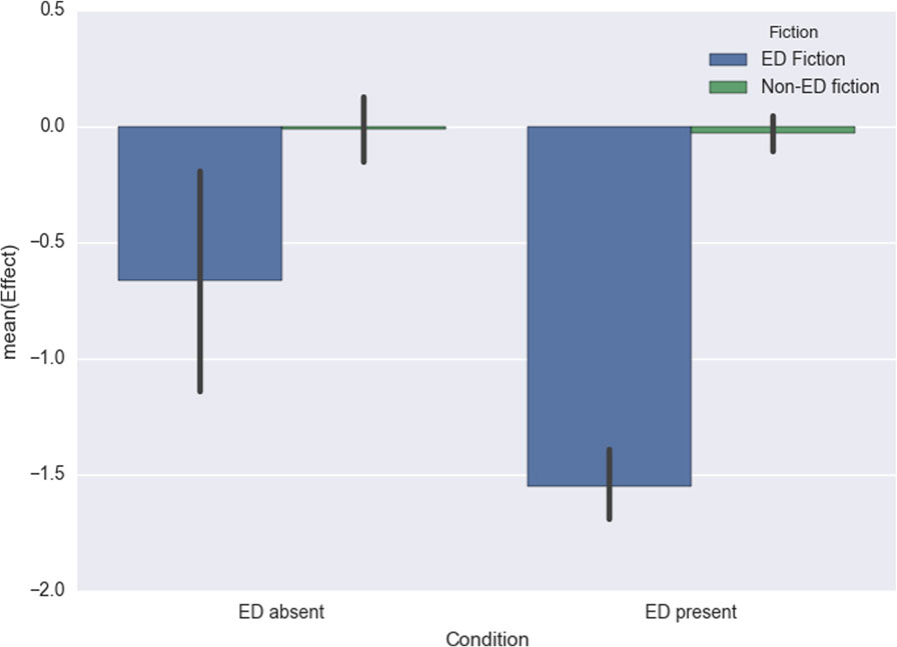
Impact on feelings about one’s body by fiction type and presence/absence of personal eating-disorder experience

Testing for Body (feelings about one’s body) showed a significant main effect for fiction type *F*(1, 262) = 54.055; *p* < .001; d = 1.055), a significant main effect for ED condition *F*(1, 262) = 10.306; *p* = .001), and a significant interaction effect between fiction type and ED condition *F*(1, 266) = 4.325; *p* < .039). Effect sizes were large and medium for the fiction type and ED conditions respectively. Effects were in the predicted direction, with ED fiction having a more negative impact on body-directed feelings (*M* = − 1.479; *SD* = 1.244) than non-ED fiction (*M* = − 0.025; *SD* = 0.631), and those with personal experience of an eating disorder being susceptible to more negative effects (*M* = − 0.788; *SD* = 1.242) than others (*M* = − 0.163; *SD* = 0.784) (Fig. 7).

**Fig. 8:**
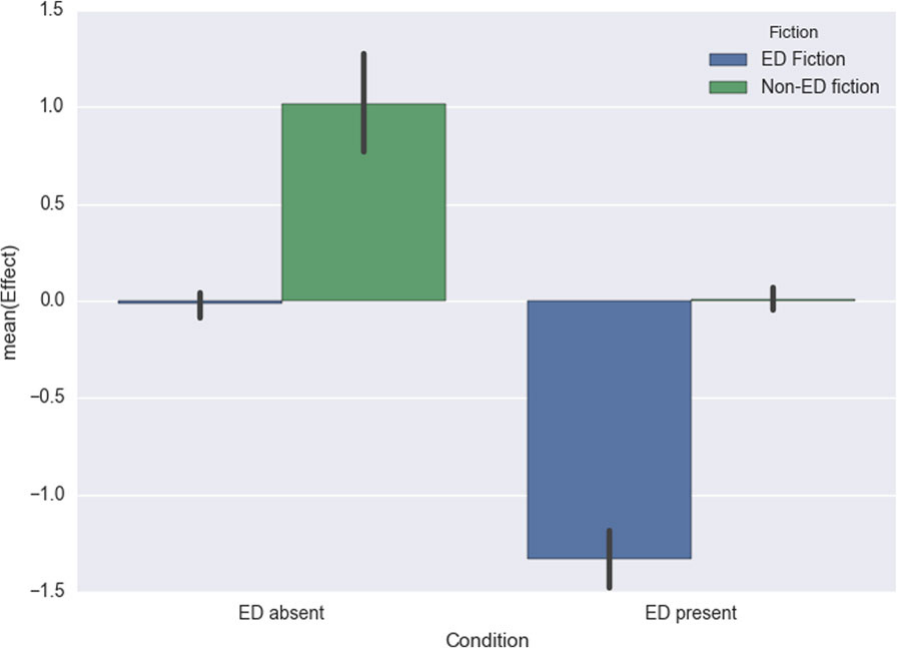
Impact on diet and exercise habits by fiction type and presence/absence of personal eating-disorder experience

Finally, the model for Diet/Exercise found significant main effects and large effect sizes for both fiction type (*F*(1, 309) = 206.110; *p* < .001; *d* = 1.08) and ED condition (*F*(1, 309) = 176.932; p < .001; *d* = 1.128). In both cases, this was in the predicted direction: ED fiction had a negative impact on diet and exercise habits (*M* = − 1.041; *SD* = 1.156) relative to non-ED fiction (*M* = 0.220; *SD* = 0.766), while those reporting a past or present eating disorder were more susceptible to negative effects with respect to diet/exercise (*M* = − 0.659; *SD* = 1.102) than those without such a history (*M* = 0.488; *SD* = 0.925). The interaction of both variables was not significant, with *F*(1, 309) = 3.416 and *p* = .06 (Fig. 8).

Comparison of effects within the group with personal eating-disorder experience was compromised by the fact that most of these respondents identified as having had more than one disorder (*n* = 153). As this comorbidity was spread across six conditions, separating out their effects is problematic. Moreover, the subpopulation of respondents who identified as having suffered from only one disorder (*n* = 121) consisted mainly of those reporting anorexia (*n* = 81). Given that the other conditions were reported in isolation by much smaller samples (binge-eating disorder = 3, bulimia = 10, compulsive overeating = 2, EDNOS = 23, other [please specify] = 2), intra-ED comparisons would have lacked statistical power and were therefore not performed.

These analyses reveal a stark difference between eating-disorder fiction and other fiction, confirming Hypothesis 1: eating-disorder fiction was found to have negative effects for both groups of readers on all dimensions except diet and exercise habits (where those with no eating-disorder history reported neutral effects), while other fiction had positive effects for both groups on the dimension of mood, and neutral effects on the other three dimensions (except for diet and exercise, where those with no eating-disorder history reported positive effects). Readers with a personal history of an eating disorder were more susceptible than others to the negative effects of eating-disorder fiction specifically on the dimensions of feelings about their body and their diet and exercise habits, partially confirming Hypothesis 2. An interaction effect between fiction type and presence or absence of an eating-disorder history was found for the dimension of feelings about one’s body.

Much can also be gleaned from what respondents wrote in the free-response sections of the survey (i.e. the invitations to elaborate on their forced-choice answers). First, their elaborations offer refinements of our main findings about the effects of fiction-reading, such as the positive effects on mood of reading non-eating-disorder fiction. Respondents identified a range of mood- and emotion-related factors as significant, including how fiction-reading improves their capacity for empathy, or calms or cheers them up. One 23-year-old recovered former sufferer said, for instance, that reading her preferred type of fiction (fantasy) ‘*Cheers me up (if it is good!) or it may inspire me. Sometimes it makes me want to grab life and live for now! Especially if I like the main character and look up to them. It can also relax me and take my mind of the daily stresses.*’

More generally, respondents bore witness to a wide range of responses to different genres in different situations: some said, for example, that reading can help distract from the pain of eating (or the pain of hunger), or exacerbate feelings of bodily inadequacy, or expand the sense of one’s place in the world and amongst other people. It can reveal the depletion of one’s powers of concentration or the nostalgia one feels for past illness, or convey to others what it feels like to have an eating disorder, or teach one tricks to exacerbate the illness. Reading can open one’s mind to different ways of being, or put one’s troubles in perspective, or offer an escape to another world, or enhance one’s emotional literacy or one’s sense of sexual possibility, or simply make one happier or calmer. In the sheer variety of these many possibilities it offers a counterpart to the typically narrower affordances of more nakedly capitalist media. As one respondent in her 60s suffering from anorexia and bulimia put it: *‘Fiction could be so valuable. We need better role models.’*

The free-response testimony also expanded on the danger of fiction about eating disorders coming to play a salient anti-therapeutic role, with 18 respondents spontaneously reporting an unexpected phenomenon: that they deliberately seek out such fiction (as well as memoirs about eating disorders) knowing that it will trigger an exacerbation of their disorder. One eating-disorder sufferer in her 30s wrote: *‘When I was younger I actively sought out these books [ED fiction] and used as deliberately triggering/pick up tips – or guide my fantasy of being 'really sick' with AN. I no longer use them or read for this reason – I want recovery and no longer find this kind of thing triggering – just sad.’* Alternatively, the text-cued exacerbation can catches one unawares: one current sufferer in her late 20s reported that *‘Reading bios I often pick up tricks (something will stick in my head, eg, "I enjoy violent purging" and now I silent berate myself when I purge to increase the force of the purging.)’*. The statistical analyses suggest that the worsening may be manifested on all four response dimensions investigated, but that those with eating disorders may be especially vulnerable to textually prompted changes to disordered behaviours and body-directed feelings.

The free responses further suggest that the most crucial factor mediating risk is the interpretive filter involved in the eating-disorder experience: readers frequently take from a text the material that supports the disorder and ignore or downplay the rest. One current sufferer (from anorexia and EDNOS) in her early 20s described this as follows: ‘*Its hard to blame the books or the authord. I feel it’s more than your ED screens out that information about pain and suffering and focuses on the success, the control and power*.’ Another respondent in recovery from anorexia and EDNOS testified with unwitting poignancy to the capacity of the eating disorder to pull everything into its orbit: ‘*Reading the Hobbit, of course, just makes one want food*.’ These effects have obvious potential to become self-fulfilling: if you read a complex fantasy novel and all it generates is the desire to eat, or read an account of an eating disorder and take from it only the positives, the cognitive constriction is further cemented and further encounters with texts or people or situations will be interpreted in light of that narrowed-down sphere of reference.

There is, consequently, widespread testimony in the free-response data to the fact that textual engagement has the potential to intervene in the mind-body feedback loops that characterise the development and maintenance of eating disorders. As analysed in depth elsewhere [48], reading can both exacerbate unstable positive feedback loops (relating to mood, concentration, preoccupation with the eating disorder, and many other variables) and introduce stabilising negative feedback into the unstable system. One respondent whose recovery from anorexia and EDNOS had stalled before she became fully healthy bore witness to dangerous positive feedback triggered by reading fiction about eating disorders: ‘*I read the book a long time ago however found it made me more determined to lose weight. My mood tended to become more low and this cemented the desire to lose weight in order to feel better about myself*’. By contrast, an undergraduate in recovery from anorexia and EDNOS (whose preferred type of fiction was *‘familiar children’s books (*e.g. *Harry Potter)’*) described the potential of other fiction to intervene in the vicious circle: ‘*It’s a distraction so I’m not thinking about my body. Also thinking about my body is connected to low mood – I usually only do it when depressed. So cheering up means I think less/less negatively about my body*.’

The causal complexities of recursive feedback relationships are a reminder to proceed cautiously in assigning ‘helpful’ or ‘harmful’ labels to particular responses. In this vein, the free-response data suggest that some reactions can initially feel like exacerbations of the disorder, but with further processing can achieve therapeutic outcomes. One 27-year-old mental healthcare assistant and research assistant in recovery from anorexia described the nostalgia that reading about eating disorders induces in her, but also the reflective processing that accompanies the initial response:

I feel a kind of nostalgia whenever I read about (in a fiction or non-fiction context) an individual with an eating disorder. I feel like I have recovered because I am no longer underweight or restricting but at the same time it reminds me that feeling envious of characters with eating disorders is perhaps a reflection that I am not 100% better!

In response to our final open-ended question, a respondent in recovery from an eating disorder (formally EDNOS but which she described as ‘essentially recurrent anorexic episodes’) reflected on how discomfort can be the precursor to greater clarity: *‘Mood can stay the same, or just be more intense, I find it inappropriate really to say it improves or worsens, that's a very black and white way of viewing things. Reflecting more can feel uncomfortable, but can also then help me see more clearly.’* Testimony of this kind warns us against hasty demarcations of ‘positive’ from ‘negative’ effects. The balance between the two may also vary between and within genres, books, and individual readers at different times. For instance, one psychology undergraduate now recovered from anorexia testified to a stark difference between past and present reading habits: *‘I read to be imaginative and feel transported away from the world. However, when I was ill I read books about eating disorders to perpetuate the disorder and put me further into that world.’*

## Discussion

Even setting aside the specific results, the overall scale of the response to our survey is in itself an indicator of the interest and importance this subject holds for people with experience of an eating disorder. Many respondents clearly devoted significant time to reflecting on and describing their experiences, and many also took the opportunity, in our final open-ended question, to express the breadth and depth of the role (fiction-)reading plays in their lives. One respondent in recovery from anorexia, bulimia, and EDNOS wrote:

A book may inspire me, give me strength to tackle life, give me ideas about how to engage with challenging situations. It may change the colour of my thoughts, the rhythm of my breath, affect the way I speak and write for a day or so. All of those things also have consequences for my relationship with my body, but as one of a number of affects, and often implicitly*.*

Taken as a whole, the survey data support what the limited prior evidence from research, policy, and practice suggest: that engagement with literary texts can play a major role in the onset, maintenance, and recovery processes associated with the biopsychosocial (or, better, biopsychosociocultural!) realities of eating disorders. The overall confirmation of Hypothesis 1, regarding the negative effects of eating-disorder fiction, is, however, a striking rebuttal of the central plank of existing creative-bibliotherapy theory: that therapeutic effects are dependent on identification with a character cued by similarity between the reader’s and the fictional character’s situations (assuming that similarity is taken to include the central fact of suffering from an eating disorder). These findings suggest the opposite: that reading about someone else who has an eating disorder may be experienced as harmful rather than helpful with respect to mood, self-esteem, body-directed feelings, and diet and exercise habits by respondents both with and without personal eating-disorder experience. Those without personal experience perceived themselves as susceptible on three of the four dimensions (not including diet and exercise habits), and reported less strongly negative effects than others on the dimensions of feelings about their body. Conversely, reading about scenarios which have nothing to do with eating disorders was perceived by both groups as having positive effects on mood and self-esteem, neutral effects on body-directed feelings, and neutral or (for those without personal experience) positive effects on diet and exercise habits. In rating texts’ helpfulness/harmfulness for their eating disorder, respondents overall also reported their preferred type of non-eating-disorder fiction to have significant therapeutic potential combined with lower anti-therapeutic potential than that associated with other text types, with positive changes in cognitive perspective particularly prominent.

Of the four dimensions of response to fiction-reading explored in detail, the dimension of mood was most strongly affected in a positive direction. The significance of mood and emotion supports their central place in the existing theory of creative bibliotherapy, but it does not mandate the assumption that only resolutely optimistic texts in which all problems are happily resolved are capable of inducing these emotionally positive effects. Some respondents mentioned, for example, that successfully meeting the challenge of reading something difficult made them feel better about themselves. One 37-year-old out of work because of anorexia and EDNOS said: *‘I feel like I have achieved something by getting through the story (concentrating is difficult for me).’* Another respondent in recovery from anorexia spoke of how helpful it is to be given, through reading, *‘perspective on the enormity/complexity of the world’*. This is far from a simplistic understanding of easy, happy texts making for happy readers.

The differentiations between the more cognitive-emotional and the more behavioural facets of response (for example, distinct effects observed for mood versus diet and exercise habits) resonate with findings which suggest that psychological aspects of disordered eating are targeted more effectively than behavioural aspects by self-help books [8]. They also offer a point of contact with theoretically geared research on health message effectiveness which uses construal level theory to frame texts at different construal levels from abstract to concrete, in order to target either (higher-level) emotion-focused or (lower-level) problem-focused coping [49]. In the eating-disorder context the importance of shifting at different points in recovery between different levels of intervention (changing specific eating habits versus developing a more acceptance attitude to one’s body, for example) may be supported by texts which create different degrees of psychological distance (by varying temporal distance in historical versus contemporary fiction, for instance, or heightening spatial and hypothetical distance in science fiction) and so prompt engagement at different levels of concreteness versus abstraction.

The survey data also shed light on an obvious but often-neglected fact in the bibliotherapy domain: that given both texts and human minds are complex, the effects of any kind of reading may be negative as well as positive. Some readers even reported deliberately looking for books to exacerbate their disorders. The possibility that reading might do harm as well as good has been broached by some researchers, considering both the text and the reader side of the interaction: the wrong book might offer pessimism or bad coping strategies [18] or simplistic answers [50], or a reader might come to a book with unrealistic expectations or an inclination to misinterpret or shirk responsibility [19]. But as I suggested above when discussing the ultimately positive emotional outcomes of not uncomplicatedly positive experiences, such dangers need not entail support for the narrow conception of what count as therapeutic texts and responses espoused by these researchers. Indeed, some risks may be the precursors to benefits, as would be suggested by a textual exposure-therapy framework. Positives and negatives may therefore not be straightforwardly separable. We need to take into account the possibly complex relationships between maximising the capacity of reading to do good and minimising its capacity to do harm, remembering that the balance may shift at different phases in an illness or a life. We also of course need to complement observational studies with experiments that investigate causality independent of self-report.

Questions about the relation between direct self-report and more indirect ways of measuring textual response are also raised by the fact that eating-disorder memoir received the highest harmfulness rankings of any fiction or nonfiction genre. This result offers a contrast not only to received wisdom in creative-bibliotherapy theory, but also to findings from a study on responses to autobiographical texts about eating disorders versus control memoirs [51], which found little effect of the eating-disorder texts on healthy undergraduates’ eating attitudes, drive for thinness, or associations between anorexia and danger or glamour. Another obvious focus of further investigation here are the differences between clinical and nonclinical populations in responses to genres like memoir.

Here it is worth noting that some respondents’ mentions of specific memoir/fiction titles made clear that the distinction between fact and fiction was not consistently applied: for example, some memoirs (e.g. Marya Hornbacher’s *Wasted*, or Grace Bowman’s *Thin*) were mentioned as examples in answers about ED fiction. Given also that a significant proportion of respondents reported harmfulness and helpfulness for both eating-disorder memoir and eating-disorder fiction (77% versus 50% for harmfulness; 73% and 64% respectively for helpfulness), this kind of blurring between categories alerts us to the possibility that the fact–fiction distinction may in some respects be a misleading one. Therapeutically speaking, reading about people with eating disorders may elicit similar responses from readers regardless of the framing of them as fictional or factual, whether because readers ignore the framing or because even when cognitively salient to readers, it is irrelevant to health-related processing and outcomes. This is suggested, for example, by the finding that transportation into a story and resulting changes to story-consistent beliefs and favourable evaluations of protagonists were unaffected by labelling that story as fact or fiction [52]. This in turn raises the question of whether some other distinction might be a stronger predictor of distinct (therapeutic or anti-therapeutic) readerly effects than fact–fiction. Other possible demarcations and dimensions include narrative versus discursive and prose versus poetic text, or the density of specific linguistic characteristics often associated with literary or poetic forms, such as figurative richness, abstraction, or other foregrounding features with defamiliarising effects [34, 35]. It also remains possible that the fictionality factor is relevant (after all, memoir and fiction were not rated identically for helpfulness or harmfulness), but that its predictive force operates in combination with another factor or factors. Probably the answer will be to some extent dependent on eating-disorder type, reader characteristics, and other contextual factors.

Pending a fuller understanding of the interactions of these textual, readerly, and contextual variables, our survey respondents’ ratings do suggest that caution must be exercised when using any textual materials that deal explicitly with eating-disorder themes. The high overlap in rates of reported help and harm provides further evidence that the same text or text type can elicit positive or negative effects in the same person at different times. Therapeutic use of eating-disorder memoirs seems to be widespread, but this may come with risks as well as benefits. These may be attributed in part to the fact that most such memoirs (like their fictional counterparts) devote significantly more space to describing the ins and outs of the illness than to charting the complexities of recovery or the contrasts and continuities between illness and life after recovery.

Further complexities arise from the ease with which a strongly pathological interpretive filter can result in highly selective readings of texts (for instance, registering nothing but food-related material in a text about many other things, or taking a pro-ED message from a text which presents both sides), and with which these in turn can generate interpretive vicious spirals. This phenomenon is one manifestation of a salient recurring feature in the free responses: namely feedback structures, and in particular the instability inherent in positive feedback loops, which are a primary characteristic of the eating-disorder psychopathology taken as a whole [37]. I have explored elsewhere the contribution these survey data make to our understanding of feedback relationships in reading and disordered eating [48], and these findings resonate with a growing interest in feedback-based predictive processing as a fundamental feature of human cognition [53]. Further research in this direction promises leverage on the dynamics of literary response as they directly impact on (and are impacted by) readers’ physical and mental health.

Beyond the precarious clinical realm, the survey findings further suggest that even readers with no eating-disorder history can be negatively affected by reading about eating disorders on the dimensions of mood, self-esteem, and (to a lesser extent) body-related feelings. However, those negative effects may not extend as far into the more fully embodied domains of changed bodily self-evaluation or behaviours around diet and exercise as may happen for those with personal experience of an eating disorder. Positive effects were also present for the group with no history of an eating disorder as a result of reading their preferred type of fiction, on the same dimensions as for the eating-disorder group: mood and self-esteem. Surprisingly, for the group without personal experience, positive effects were also reported on the diet and exercise dimension. Together these results suggest the possibility of a preventative or positively health-enhancing role for recreational reading in the general population. The wide range of literary genres reported as eliciting positive effects also encourages us to look beyond the narrow confines of realistic, problem-orientated, optimistic fiction recommended in the few existing theoretical models.

Finally, the interaction effect between fiction type and reader group solely on the dimension of body-directed feelings implies that the body and how it is experienced and appraised may act as a fulcrum between the text- and reader-driven facets of response. As a midway point between generalised mood and self-esteem and the body-orientated actions of eating and exercising, feelings about one’s body may be a critical locus at which to observe how readers’ personal history affects their responses to specific textual features. This aligns with the accumulating evidence for the contributions of embodiment to the whole spectrum of what we may think of as cognitive, emotional, or perceptual activity [54].

These reflections need qualifying by mention of some obvious limitations of the present research, the first being that it is wholly observational: it offers no way of separating correlation from causation, so there may be significant confounding factors (such as severity of illness, or motivation for recovery) at play in the results. Second, the results are limited to verbal self-report: to respondents’ retrospective assessment of the relationships under investigation. The highly significant trends revealed in the statistical analysis may in part reflect the fact that respondents report what they *think* the effects of reading have been, rather than direct effects of exposure to certain text types. Because the reported experiences and effects were in the past, we were also unable to use any validated measures of mood, self-esteem, or eating/body-related variables to assess their extent and composition more fully. Beyond the usual limits and distortions that may be present in anyone’s insight into the causal relationships at play in their own life, self-report as specifically retrospective act is significant here in that we did not ask respondents to specify at which point in the past any given experience occurred. This means that although we have information, for example, about their current recovery status, we do not know whether a reported reading experience took place when that status was different, although this can often be easily inferred in individual cases from the additional comments provided. The phrasing of the questions was in the present tense, i.e. prompting focus on current tendencies, but it is clear that some respondents sometimes referred to events in the relatively distant past, when various physical or psychological factors may or may not have been different.

Another set of complexities relate to the variety of eating-disorder types reported by respondents. Given the unequal numbers of respondents reporting experience of anorexia versus other eating disorders, much of the statistical variance in the group with personal experience was probably driven by anorexia, and more research is needed on how the individual eating disorders affect responses to textual materials. For simplicity’s sake, we also allowed respondents to report the disorder(s) they had suffered from and their current recovery status with no reference to formal diagnosis; we considered widespread misreporting or ‘fabrication’ of such illness experiences highly unlikely, and also considered it important to trust respondents to assess their own degree of recovery (especially given the pragmatic constraints which encourage many treatment providers to set unrealistically low thresholds for ‘recovery’: there is no infallible arbiter of recovery). Finally, respondents were self-selecting, so they may represent a subpopulation (albeit a sizeable one) of people for whom reading is especially significant. In particular, due to limitations on how the survey was publicised, the sampling method for the more general population of respondents with no personal history of disordered eating cannot confidently be considered representative (indeed, nearly half reported having a family member with a past or present eating disorder), and any conclusions drawn should reflect this limitation.

Overall, however, future research and practice in this area must proceed on the basis of unidealistic acceptance of the real potential of reading to do harm as well as good, in ways dependent on multiple aspects of the text, the reader, and the context of reading. Future work will build on the results presented here via a randomised controlled trial using validated outcome measures to investigate the efficacy of creative bibliotherapy for eating disorders, in conjunction with shorter mechanisms studies to complement the main-effect trial. This will give us purchase on the interactions of reader and text variables at play in therapeutic reading, and promises insights into key questions that arise from our preliminary findings. These include: how best to work therapeutically with readers’ interpretive filters; how to balance short-term discomfort with longer-term good; how to harness positive and negative feedback to therapeutic effect; which dimensions of character-reader similarity and difference are most relevant; and whether the crucial variations in textual effects are best captured by the spectrum from fiction to nonfiction or from narrative to discursive or prose to verse forms, or by any other set of generic or stylistic markers.

## Conclusion

There are many possible reasons why the relevance of literary reading to eating disorders has been overlooked, from the one extreme of not believing that art could *really* be relevant to health to the other of wanting to believe in rather than test the healing power of literature. But the rich and suggestive testimony gathered in this survey makes clear that literary reading can play crucial roles for those suffering from mental illness, right across the spectrum from serving as an indispensable everyday tool (‘*Sometimes when I am tempted to restrict I read for half an hour before a meal to get myself into the mood* [for eating]’) to opening up possibilities for imagining that life really could be otherwise:

I often find it difficult and/or scary to express how I feel in words or to others. Books and in particular fiction provide a kind of transitional and creative space for that – I can relate to aspects of myself (both similar and different) in the voices of the characters, their journeys and so on – and offer an opportunity to go beyond my experience and explore different possibilities, ideas, and so on, without completely disconnecting from them. I find reading completely invaluable as a therapeutic experience and means of exploring my feelings and potential.

The results of this survey suggest a highly significant divergence between disorder-specific fiction (in which, for example, a main character suffers from the same condition as the reader) and readers’ preferred genre of other fiction: eating-disorder fiction is typically perceived as detrimental on dimensions central to the experience of an eating disorder (mood, self-esteem, feelings about one’s body, and diet and exercise habits) while other fiction is often perceived to have positive effects, especially on mood. This finding directly contradicts existing theoretical models of creative-bibliotherapeutic effects, all of which emphasise reader–character similarity, and it offers a complement to existing empirical work on self-help bibliotherapy, which is as yet theoretically underdeveloped.

Concerted interdisciplinary investigation will be needed to pin down the cognitive-linguistic interactions at play in any textually mediated therapy: how individual words and phrases are likely to shape a given reader’s response in a given context. The indications presented here of the double-edged power of literary reading suggest that there is much to be gained from developing our understanding of the role of textual interpretation within the complex mind-body system that is an eating disorder.
